# A Systematic Approach for Multidimensional, Closed-Form Analytic Modeling: Minority Electron Mobilities in Ga_1−x_Al_x_As Heterostructures

**DOI:** 10.6028/jres.105.037

**Published:** 2000-06-01

**Authors:** Herbert S. Bennett, James J. Filliben

**Affiliations:** National Institute of Standards and Technology, Gaithersburg, MD 20899-0001

**Keywords:** electron mobilities, melding functions, regression analyses, standard deviations

## Abstract

A significant, practical challenge, which arises in developing computationally efficient physical models for use in computer simulations of microelectronic and optoelectronic devices (for example, transistors in digital cellular phones and lasers in optical networks, respectively), is to represent vast amounts of numerical data for transport properties in two or more dimensions in terms of closed form analytic expressions. In this paper, we present a general methodology to achieve the above goal for a class of numerical data in a bounded two-dimensional space. We then apply this methodology to obtain a closed-form analytic expression for the minority electron mobilities at 300 K in p-type Ga_1−_*_x_*Al*_x_*As as functions of the acceptor density *N*_A_ between 10^16^ cm^−3^ and 10^20^ cm^−3^ and the mole fraction of AlAs *x* between 0.0 and 0.3. This methodology and its associated principles, strategies, regression analyses, and graphics are expected to be applicable to other problems beyond the specific case of minority mobilities addressed in this paper.

## 1. Introduction and Motivation

Researchers often first obtain theoretical and/or experimental results in the form of multi-dimensional, numerical tables consisting of discrete data-points and then use families of traces to represent graphically their discrete data-points in a more easily understood manner. Such simplified graphical representations are a common recourse when several complex and competing physical mechanisms occur and when multidimensional, closed-form analytic expressions are not available. Incorporating such discrete data tableaux into physical models for use in computer simulations is usually not satisfactory due to excessive computer time associated with interpolations between the discrete data points. Since such computational inefficiencies associated with “look-up” tables occur, industry is reluctant to incorporate them in semiconductor device simulators that run on engineering workstations. This is particularly the case when numerical differences must be used to compute first and higher order derivatives.

In this paper, we present a general strategy based on separable functions, melding functions, transformations, admissible non-linear methods, and regression analyses to obtain multi-dimensional, closed-form analytic expressions from tables containing discrete data points. As an illustrative example, we apply this general strategy to a class of numerical data in a bounded two-dimensional space and show how to obtain a two-dimensional, closed-form analytic expression for the minority electron mobilities in p-type Ga_1−_*_x_*Al*_x_*As. The acceptor density *N*_A_ varies between 10^16^ cm^−3^ and 10^20^ cm^−3^ and the mole fraction of AlAs *x* varies between 0.0 and 0.3. By so doing, we respond to the need for predictive computer simulations of devices that have fewer unknown or variational parameters [1].

Many device simulators for bipolar and field-effect transistors require a variety of physical models and associated input parameters to describe fully how carrier transport varies with carrier concentrations, ionized dopant densities, alloy mole fractions, and temperature. This paper focuses on one such model, namely, the model for how the minority electron mobility varies with dopant density and mole fraction of AlAs at 300 K. Self-consistent numerical solutions to the quantum mechanical, non-linear integral-differential equations for carrier transport in semiconductors result in discrete data-points that by themselves do not readily suggest closed-form analytic expressions for transport properties, carrier densities of states, and band structure changes. Interpolating among the discrete data-points in “look-up” tables leads to discontinuities, particularly in numerical derivatives, and, as mentioned above, is computationally inefficient.

The motivation for our performing the following analysis is to derive a closed-form analytic expression that will result in more efficient computer simulations and improved physical insights on how the many scattering mechanisms, which influence carrier transport in ternary compound semiconductors and heterostructure devices, affect their electronic and optical behavior. Our data analysis, presented in the following sections, enables us to reduce the number of unknown physical parameters in numerical simulations that predict electrical and optical performance of devices such as bipolar transistors, solar cells, laser diodes, and light-emitting diodes. The latter are used to read digital versatile disks (DVD).

The development of such improved closed-form mobility models directly impacts the design of microwave heterojunction bipolar transistors used in the linear power amplifiers of digital cell phones. The design challenges and goals are low noise and very linear, efficient power amplifiers. Such amplifiers enable longer talk times and improved adjacent-channel rejection in the dense channel packing that is necessary to maximize the capacity of communications systems. Designers of these amplifiers rely, in part, on device simulators to give them physical insights for selecting the optimum technology and designing reliable, low cost devices. Using device simulators also provides sources of expert knowledge from others, shortens the time to market by reducing the number of experiments needed for design verification, and saves money. In order to increase the likelihood that computer simulations of heterostructure devices such as heterojunction bipolar transistors (HBTs) will be predictive and thereby more useful for the future development of HBTs, it is essential to have accurate values for the minority mobilities of electrons in Ga_1−_*_x_*Al*_x_*As. This is especially true when one designs the bases of HBTs in mobile phones that use code division multiple access (CDMA) protocols. Such protocols require very linear power amplifiers for acceptable adjacent-channel rejection. These amplifiers also should consume a minimum of energy. Device manufacturers may then have greater confidence when using more predictive simulators to design products.

The markets for HBTs in mobile and lightwave communications systems are increasing [2–6]. For example, the worldwide number of new, cellular subscribers exceeded the number of new subscribers for conventional wired telephones or fixed networks for the first time in 1995 [7]. Market demands for telecommunications and optical storage systems that contain laser diodes (LDs) and light-emitting diodes (LEDs) offer many opportunities for ternary III–V compound semiconductor HBTs. A common feature for most of these devices in such systems is that they contain active regions with high concentrations of dopants and carriers. Such high concentrations alter the densities of states for the carriers and the band structure of the semiconductors. These in turn greatly affect how carriers move in semiconductors and modify substantially carrier transport parameters such as carrier mobilities, lifetimes of carriers, and effective carrier concentrations. Accurate physical models for these transport parameters are needed in device simulators so that they may be more predictive when manufacturers use them to design products.

## 2. Data Table for Minority Electron Mobilities in p-type Ga_1−_*_x_*Al*_x_*As

This section summarizes the background details by which the data table for minority electron mobility values was obtained. This data table serves as our starting point for deriving the closed-form analytic expression for the mobility model.

The theoretical calculations in Ref. [8] include all the important scattering mechanisms for the low-field mobilities of electrons in heavily doped Ga_1−_*_x_*Al*_x_*As: acoustic phonon, polar optic phonon, nonpolar optic phonon (holes only), piezoelectric, ionized impurity, carrier-carrier, plasmon scattering, and alloy scattering. The Boltzmann transport equation is solved by the variational procedure outlined by Walukiewicz et al. [9]. This method avoids the use of the relaxation-time approximation that is invalid for mechanisms that involve energy transfers comparable to or greater than *k*_B_*T*, where *k*_B_ is the Boltzmann constant, and *T* is thermodynamic temperature. Also, Matthiesson’s rule is not used because it is not valid for Ga_1−_*_x_*Al*_x_*As, as shown in Ref. [9]. The scattering rates are summed prior to the variational solution. The results are highly accurate calculations of the minority mobilities for p-type Ga_1−_*_x_*Al*_x_*As at 300 K as functions of the dopant density and the mole fraction of AlAs. The calculated results agree well with the rather limited experimental data for Ga_1−_*_x_*Al*_x_*As mobilities [10–12].

The calculations were done for a full factorial design consisting of 21 discrete values of acceptor density *N*_A_ between 10^16^ cm^−3^ and 10^20^ cm^−3^ and seven discrete values of mole fraction *x* between 0.0 and 0.30, namely, *x* = 0.0, 0.05, 0.10, 0.15, 0.20, 0.25, and 0.30 [denoted also by *x* = 0.00 (0.05) 0.30, a general notation that we use latter on in this paper], to yield a total of 147 data points. We use the notation that *x*_1_ = 0.00, *x*_2_ = 0.05, … *x*_7_ = 0.30, respectively. The self-consistent, numerical solutions to the quantum mechanical, non-linear integral-differential equations for carrier transport in semiconductors are given in Table 1 as a two-dimensional array of discrete data-points. This data representation, as opposed to a functional representation, was necessary because the several competing scattering mechanisms, listed above and considered all together, do not readily yield any acceptable theoretical closed-form analytic expression. Over 90 h of NIST Cray YMP supercomputer^1^ time were used to obtain these 147 data points.

**Fig. 1:**
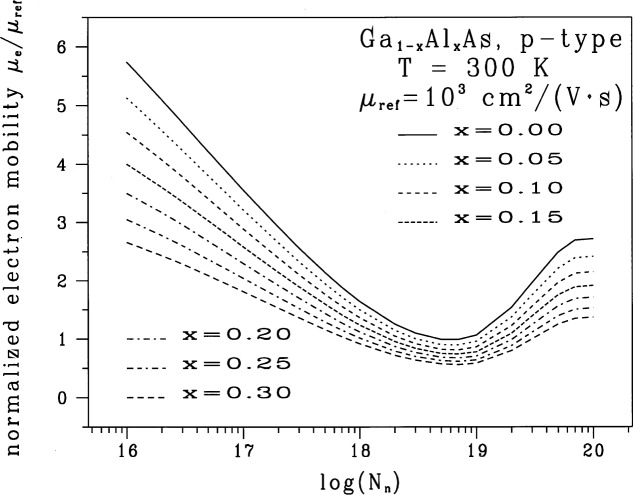
Minority electron mobility in p-type Ga_1−_*_x_*Al*_x_*As for several values of mole fraction in the range 0.0 ≤ *x* ≤ 0.3. These curves include the effects of the electrons scattering off hole plasmons and of deducting the density of holes with energies below the Fermi energy from the electron-hole scattering process [8]. The plasmon cut-off factor (PCF) $q_{c}^{2}r_{s}^{2}$ is 1 where *q*_c_ and *r*_s_ are the cut-off wave number and the screening radius, respectively. The mobilities have been normalized to *μ*_ref_ = 1000 cm^2^/(V·s). The dimensionless acceptor density is *N*_n_ = *N*_A_ cm^3^.

The 147 data points presented in Table 1 are represented graphically in Fig. 1 as a family of seven traces corresponding to the seven mole fraction values *x* = 0.00 (0.05) 0.30, respectively. The fixed increment of *x_i_*_−1_−*x_i_* = 0.05 for all *i* and a subsequent fortuitous response surface in the mole-fraction variable will be advantageously employed later to simplify the fitting process.

We thus have the task of finding a closed-form two-dimensional analytic function *g* for the minority electron mobility *μ*_e_ in p-type Ga_1−_*_x_* Al*_x_* As such that *μ*_e_ = *g*(*N*_A_, *x*). For simplicity we may alternatively find a function *f* for the dimensionless normalized mobility *Y* = *μ*_e_/*μ*_ref_ such that

$$Y=f\left(N_{A},x\right)$$ (1)

where *g* = *μ*_ref_ *f* and 2 ref *μ*_ref_ = 1000 cm^2^/(V · s). To achieve an acceptable analytic fit of *Y* to *N*_A_ and *x* for use in semiconductor device simulators that run on engineering workstations we require a relative residual standard deviation for *Y* that is less than 2 %.

The development of such a function *f* would represent a significant increase in computational efficiency by about a factor of 5 and gives mobility models for use in commercial semiconductor device simulators that are in closer agreement with measurements. The combination of the existing NIST supercomputer-generated mobility data and the derived two-dimensional analytic function *f* will lead to computer simulators that are at once both more parsimonious (have fewer unknown or tuning-variational parameters) and more accurate (offer improved predictability).

## 3. Data Analysis for Minority Electron Mobilities in p-type Ga_1−_*_x_*Al*_x_*As

In the following sections we show that using a combination of separable functions, melding functions, transformations on the discrete data points in Fig. 1, and non-linear regression analyses leads to a single two-dimensional, closed-form analytic expression for the minority electron mobilities at 300 K in p-type Ga_1−_*_x_*Al*_x_*As as functions of the mole fraction of AlAs *x* between 0.0 and 0.3 and the acceptor density *N*_A_ between 10^16^ cm^−3^ and 10^20^ cm^−3^. Throughout our analyses we rely substantially on graphics and keep the number of fitting coefficients to a minimum, subject to the constraint that the residual standard deviation *S*_res_ (*Y*) as defined by Eq. (2) satisfies *S*_res_(*Y*) ≤ 0.02. The residual standard deviation is a measure of the “average” error in a fitted model and thereby is a metric for assessing the quality of the fit, with a smaller *S*_res_(*Y*) indicating a better fit. The residual standard deviation for a model *Y* = *f*(*N*_A_, *x*) is

$$S_{\text{res}}\left(Y\right)=\sqrt{\left[\sum_{j=1}^{n}{\left(Y_{j}-\bar{Y_{j}}\right)}^{2}/(n-p)\right]}$$ (2)

where *Y_j_* are the observed data values, the $\bar{Y_{j}}$ are the predicted values from the fitted model, *n* is the total number of data points (here *n* = 147) and *p* is the total number of parameters to be fitted in the model (here *p* = 18). The residual standard deviation for a model, Eq. (2), with *p* parameters differs from the standard deviation for a set of data points *S*_D_(*Y*). The standard deviation for a set of data points is given by

$$S_{D}\left(Y\right)=\sqrt{\left[\sum_{j=1}^{n}{\left(Y_{j}-\bar{Y}\right)}^{2}/(n-1)\right]}$$

where $\bar{Y}=\sum_{j=1}^{n}Y_{j}/n$ is the arithmetic average.

We use the NIST-developed DATAPLOT [13] software for both the exploratory graphics and for the extensive non-linear statistical analyses. Also, for those cases in which the residual standard deviations from analyses based on different functional forms are quantitatively similar, we select the functional form that will minimize the computer time when the closed-form analytical function is used in commercial simulators and select procedures that have a minimum of fitting parameters.

Our general strategy is based on separable and melding functions and on transformations of the response function *Y* that give near-linear separable functions as described below. We want to obtain the function *Y* = *f*(*X*, *x*) in the two-dimensional continuum space spanned by *X* = log[*N*_A_/(10^16^ cm^−3^)] and *x*. This bounded two-dimensional continuum is given in Fig. 1 with 0 ≤ *X* ≤ 4 and 0.00 ≤ *x* ≤ 0.30. As with fitted functions, extreme caution must be exercised in extrapolating beyond these *X* and *x* limits.

### 3.1 Separable Functions

Consider the discrete two-dimensional space given by the 147 data points in Fig. 1. Let *Y_i_* denote the *i*th data trace in Fig. 1 with *i* = 1(1)7 corresponding to mole fraction *x* = 0.00(0.05)0.30. *If* the *Y_i_* are related to *Y*_1_ via expressions of the form

$$Y_{i}=H_{i}\left(Y_{1}\right)$$

and *if Y*_1_ is related to *X* by

$$Y_{1}=B\left(X\right)$$

then *Y_i_* = *H_i_*[*B*(*X*)].

In practice, *H_i_* and *B* are least-squares, best-fit functions relating *Y_i_* to *Y*_1_ and *Y*_1_ to *X*, respectively. We call the functions *Y_i_* and *B* separable functions. In addition, if the *Y_i_* were to be *linearly* related to *Y*_1_, that is, if

$$Y_{i}=H_{i}\left(Y_{1}\right)=I_{i}+S_{i}Y_{1},$$

where *I_i_* and *S_i_* denote the *i*th intercept and the *i*th slope, respectively, then

$$Y_{i}=H_{i}[B(X)]=I_{i}+S_{i}B(X).$$

In this case, *Y_i_* and *B* are called linearly separable functions.

We now determine the extent to which the raw data in Fig. 1 may be represented by separable and by linearly separable functions. Without any additional information besides that contained in Fig. 1, we could select any one of the seven traces as a base trace and examine how the other six traces relate to the base trace. However, since we know that trace *Y*_1_ for GaAs (the *x* = 0.0 trace in Fig. 1) has been verified experimentally by three separate groups [10–12], we can use with no loss in generality, trace *Y*_1_ as the reference or base trace.

We now discuss the strategy for determining the nature of the relationship *H_i_* between the trace *Y_i_* and the base trace *Y*_1_. We tentatively hypothesize that the trace *Y_i_* is linearly related to *Y*_1_. We would thus have the 12 parameter representation for *i* = 2(1)7,

$$Y_{i}=H_{i}\left(Y_{1}\right)=I_{i}+S_{i}Y_{1},$$ (3)

with six values of intercepts *I_i_* and six values of slopes *S_i_*. The graphical analog for Eq. (3) is a fan-shaped collection of traces in the *Y_i_* vs *Y*_1_ plot.

The first simplification for Eq. (3) would be for all six slopes *S_i_* to be identically equal to a constant slope *S*; thus, yielding the seven parameter representation

$$Y_{i}=I_{i}+SY_{1}.$$ (4)

Graphically, Eq. (4) results in a *Y_i_* versus *Y*_1_ plot consisting of seven parallel lines.

The second simplification would be for the six intercepts to be a constant multiple of *i* as in *I_i_* = *I* + (*i−*1)*D*, where *I* and *D* are constants, thus yielding the three parameter representation

$$Y_{i}=I+\left(i-1\right)D+SY_{1}.$$ (5)

Graphically, Eq. (5) results in a *Y_i_* vs *Y*_1_ plot consisting of seven parallel lines having constant spacing.

**Fig 2:**
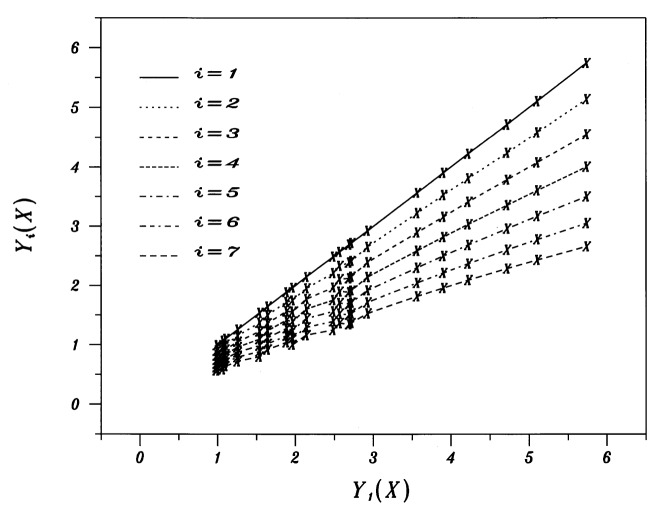
Plots of *Y_i_*(*X*) vs *Y*_1_(*X*) for *i* = 1(1)7.

Examination of Fig. 2 shows that the seven traces of *Y_i_* vs *Y*_1_ for the mobility data are not perfectly parallel and do not exhibit fixed spacing. This indicates that the simple three parameter representation is not adequate. We note from Fig. 2 that the family of seven traces do exhibit rough linearity with different intercepts *I_i_* and with markedly different slopes *S_i_*, thereby tenuously justifying our 12 parameter linear representation. At this point a series of six linear fits could be carried out from Fig. 2, thus yielding estimates for the 12 parameters (six intercepts and six slopes).

We did *not* do this for the following four reasons:

1. Non-linearity: From Fig. 2, we note that because the traces exhibit subtle but progressively increasing curvature for *i* = 2(1)7, the linearity assumption is violated.
2. Parsimony: Parsimony dictates that the number of parameters be as small as possible and it is preferable to reduce the 12 parameters to a smaller number.
3. Stability: Estimating fewer parameters is usually computationally more stable.
4. It may be possible to find a representation for the p-type minority electron mobilities that is more fundamental and hence may be applicable to other semiconductor modeling cases.

For the reasons cited above, we set aside the 12 parameter representation and focus on a modified (and ideal) three parameter representation. Clearly, from Fig. 2, this cannot be achieved for the original normalized values of *Y*, because

$$Y_{i}\neq I+\left(i-1\right)D+SY_{1}.$$ (6)

Our strategy is then to seek in Sec. 3.2 the transformation *U* of the mobility data such that

$$U\left(Y_{i}\right)=I+\left(i-1\right)D+SU\left(Y_{1}\right).$$ (7)

We assign the notation *Z_i_* to the transformed mobility data *U*(*Y_i_*) and obtain

$$Z_{i}=U\left(Y_{i}\right)=I+\left(i-1\right)D+SZ_{1}.$$ (8)

### 3.2 Transformation of Mobility

To determine the appropriate transformation *U* we consider the following extended power-transformation family:

$$Z_{i}=\left(Y_{i}^{\lambda }-1\right)/\lambda$$ (9)

for λ = − 1.0(0.5)1.0. When λ = 0 the transformation is the natural logarithm, namely

$$Z_{i}=\ln Y_{i}=\underset{\lambda \to 0}{\lim}[(Y_{i}^{\lambda }-1)/\lambda ].$$ (10)

**Fig. 3:**
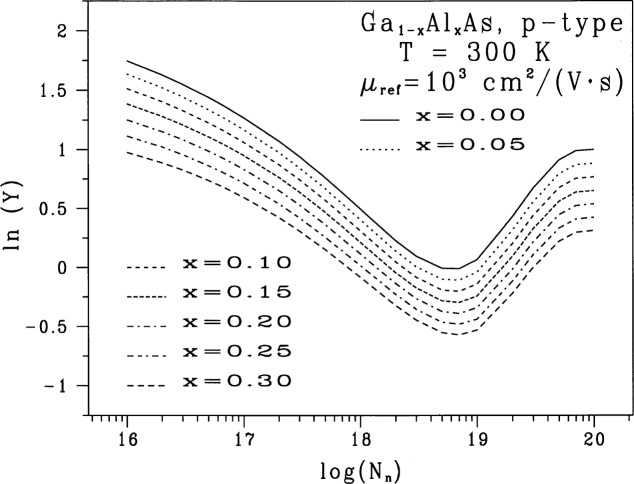
Plots of the natural logarithm of the normalized minority electron mobility in p-type Ga_1−_*_x_*Al*_x_*As for several values of mole fraction in the range 0.0 ≤ *x* ≤ 0.3. These curves include the effects of the electrons scattering off hole plasmons and of deducting the density of holes with energies below the Fermi energy from the electron-hole scattering process [8]. The plasmon cut-off factor (PCF) $q_{c}^{2}r_{s}^{2}$ is 1 where *q*_c_ and *r*_s_ are the cut-off wave number and the screening radius respectively. The mobilities have been normalized to *μ*_ref_ = 1000 cm^2^/(V·s). The dimen sionless acceptor density is *N*_n_ = *N*_A_ cm^3^.

**Fig. 4:**
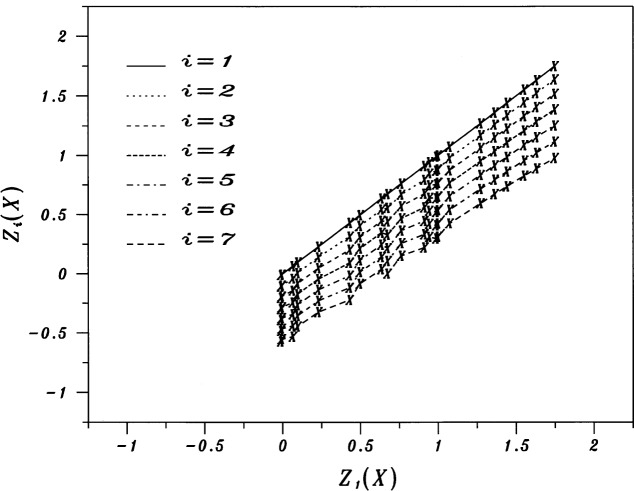
Plots of the transformed function *Z_i_*(*X*) vs *Z*_1_(*X*) for *i* = 1(1)7.

**Fig. 5:**
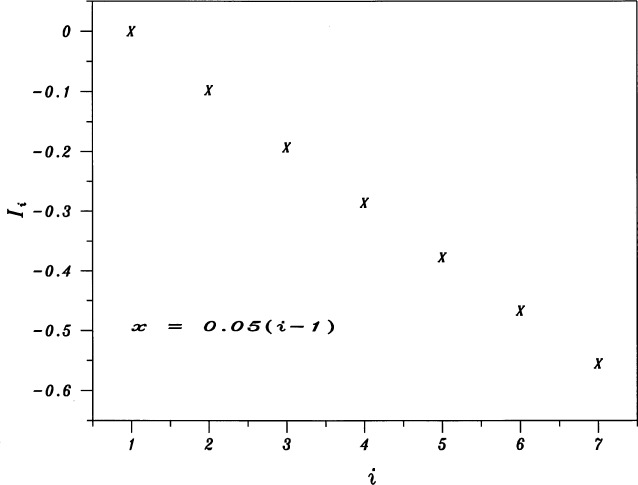
Plot of the intercepts *I_i_* for the transformed curves *Z_i_*(*X*_0_) at *X* = *X*_0_ vs *i* = 1(1)7. The mole fraction *x* is given by *x* = 0.05 (*i* 1).

**Fig. 6:**
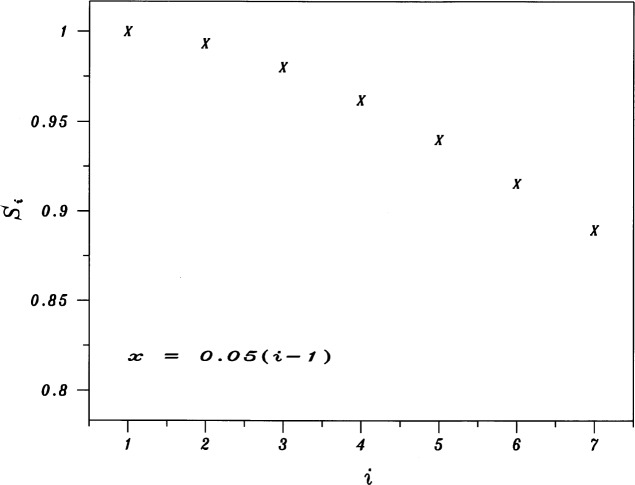
Plot of the slopes *S_i_* for the transformed curves *Z_i_*(*X*_0_) at *X* = *X*_0_ = 0 vs *i* = 1(1)7. The mole fraction *x* is given by *x* = 0.05(*i* 1).

Figures analogous to Fig. 1 for each of the five values of λ show that when = 0, the seven traces of *Z* vs *X* are the closest to being nearly parallel and equally spaced as shown in Fig. 3. Figure 4 then gives the seven traces of *Z_i_* vs *Z*_1_. The next step is to plot in Figs. 5 and 6 the intercepts *I_i_* and the slopes *S_i_* for the transformed traces *Z_i_*(*X*_0_) = ln *Y_i_*(*X*_0_) at *X* = *X*_0_ = 0 vs *i*. Polynomial fits for the intercepts and slopes give expressions of the form

$$I\left(x_{i}\right)=a_{0}+a_{1}x_{1}+a_{2}{x_{i}}^{2}+a_{3}{x_{i}}^{3}+\ldots +a_{2}{x_{i}}^{n}$$ (11)

and

$$S\left(x_{i}\right)=s_{0}+s_{1}x_{i}+s_{2}{x_{i}}^{2}+s_{3}{x_{i}}^{3}+\ldots +s_{n}{x_{i}}^{n}$$ (12)

where *x_i_* = 0.05 (*i−*1) and *i* = 1(1)7. The condition of near-constant spacing for *I_i_* means that |*a*_0_ + *a*_1_ *x_i_*| should be much greater than |*a*_2_ *x_i_*^2^ + *a*_3_ *x_i_*^3^ + … + *a_n_ x_i_^n^*|; and the condition of near-constancy for *S_i_* means that |*s*_0_| should be much greater than |*s*_1_ *x_i_* + *s*_2_ *x_i_*^2^ + *s*_3_ *x_i_*^3^ + … + *s_n_ x_i_^n^*|. The residual standard deviations *S*_res_(*I*) and *S*_res_(*S*) of polynomial fits to *I*(*x*) and *S*(*x*) are calculated for increasing orders *n* of the polynomials until acceptably small values of *S*_res_ occur. For the data in Fig. 1, cubic fits to both *I*(*x*) and *S*(*x*) give *S*_res_(*I*) = 0.000 11 and *S*_res_(*S*) = 0.000 04 in the logarithmically transformed space. These values of *S*_res_(*I*) = 0.000 11 and *S*_res_(*S*) = 0.000 04 are sufficiently small to achieve the overall goal of *S*_res_(*Y*) ≤ 0.02.

In summary, the general strategy is to replace the difficult task of finding one function *f* with three easier tasks of finding a base function *B*(*X*), an intercept function *I*(*x*), and a slope function *S*(*x*) that is weakly dependent on *x* and for which *i* is the surrogate for the mole fraction variable *x* = 0.05(*i−*1).

### 3.3 Melding Functions

The task of finding the reference or base function *B*(*X*), which is essentially the minority electron mobility for GaAs, involves the following series representation for *B*(*X*):

$$B\left(X\right)=\sum_{l=1}^{N}w_{l}\left(X\right)g_{l}\left(X\right),$$ (13)

where the weighting functions *w_l_*(*X*) satisfy the sum rule $\sum_{l=1}^{N}w_{l}\left(X\right)=1$. The function *B*(*X*) is represented by a melding function. A melding function in this case is a global *N*-region additive function that joins the *N* local functions *g_l_*(*X*) to give a smooth function by the use of the weighting functions *w_l_*(*X*) that are bounded between 1 and 0 over the global region of *X*. For the illustrative example here *X* is bounded between 0 and 4.

Figure 1 suggests that it is necessary to consider only two regions namely *N* = 2. That is, we write *B*(*X*) as

$$B\left(X\right)=w\left(X\right)g_{1}\left(X\right)+\left(1-w\left(X\right)\right)g_{2}\left(X\right).$$ (14)

Commonly used functions for the local functions *g_l_*(*X*) are polynomials of *n*th order, rational functions, spline functions, other flexible functions, and melding functions themselves. We use cubic polynomials for the functions *g_l_*(*X*), namely,

$$g_{1}\left(X\right)=c_{10}+c_{11}X+c_{12}X^{2}+c_{13}X^{3}$$ (15)

and

$$g_{2}\left(X\right)=c_{20}+c_{21}X+c_{22}X^{2}+c_{23}X^{3},$$ (16)

because they, as for the above intercepts and slopes, give acceptably small *S*_res_(*g*_1_) and *S*_res_(*g*_2_) values.

There are initially many choices for the weighting functions *w*(*X*). The requirement that *S*_res_(*Y*) ≤ 0.02 eliminates many of them. The two weighting functions that give the smallest *S*_res_(*Y*) values for the data in Fig. 1 are the normal cumulative distribution function (CDF) and the logistic CDF (LCDF). The *S*_res_(*Y*) values for the normal CDF and the LCDF are almost the same. Since the LCDF requires less computer time to evaluate, we select it for the remaining analyses. The LCDF is given by

$$L\left(X,X_{0},\sigma_{0}\right)=1/\left\{1+\exp\left[-\left(X-X_{0}\right)/\sigma_{0}\right]\right\}$$ (17)

where *X*_0_ and *σ*_0_ are location and scale parameters of the logistic distribution.

## 4. Final Results—Closed-Form Analytic Function

The function *B*(*X*) is a global function in ln*Y* space composed of the two local functions *g*_1_(*X*) and *g*_2_(*X*) that have the same value at the region boundary *X* = *X*_b_. Figure 1 suggests that *X*_b_ should be near *X*_b_ = 2.75. The next step is to obtain cubic non-linear fits for *g*_1_(*X*) over the region 0 ≤ *X* ≤ *X*_b_ and for *g*_2_(*X*) over the region *X*_b_ ≤ *X* ≤ 4.0.

The regression analyses involves three major steps:

1. Determine the non-linear base function *B*(*X*) that relates the logarithm of the reference mobility trace *Z*_1_ directly to *X*.
2. Determine the intercept function *I_i_* and the slope function *S_i_* that relate the other logarithms of the mobility traces *Z_i_* to *Z*_1_. In effect this allows the mole-fraction variable *x* to enter the fit by means of the relation *x* = 0.5(*i−*1). At this point, we have adequate non-linear fits relating the *Z_i_* to the doping density *X* and the mole-fraction *x* via the expressions *Z_i_* = *H_i_*[*B*(*X*)] = *I_i_* + *S_i_*[*B*(*X*)].
3. Use the estimated fitting coefficients from Step 1 above for the base function fitting [namely *c*_1_*_j_*, *c*_2_*_j_*, *X*_0_ and *σ*_0_, where *j* = 0(1)3] as initial values for the final non-linear fit relating the mobilities *Y_i_* to *X* and *x* by means of the function

$$Y_{i}=\exp[Z_{i}]=\exp\{H_{i}[B(X)]\}=\exp[I_{i}+S_{i}B(X)].$$ (18)

For this final fit, only the 10 base function coefficients *c*_1_*_j_*, *c*_2_*_j_*, *X*_0_ and *σ*_0_ are varied. There is no need to vary again the intercept and slope coefficients *a_j_* and *s_j_* from Step 2.

Combining all of the numerical and statistical procedures described in Steps 1 to 3 above, we obtain a two-dimensional closed-form analytic expression for electron mobilities in p-type Ga_1−_*_x_*Al*_x_*As as functions of acceptor density *N*_A_ and mole fraction *x* of AlAs, namely,

$$\begin{array}{c} \mu_{e}\left(\text{p-type};N_{A},x\right)=\mu_{\text{ref}}\exp\{I\left(x\right)\\ +S\left(x\right)[w_{L}\left(X\right)g_{1}\left(X\right)+\left(1-w_{L}\left(X\right)\right)g_{2}\left(X\right)]\} \end{array}$$ (19)

where *N*_A_ = 10*^X^* × 10^16^ cm^−3^, where the seven traces containing 147 data points in Fig. 1 are represented by the four cubic polynomial functions *I*(*x*), *S*(*x*), *g*_1_(*X*), and *g*_2_(*X*), and the exponential type function *w_L_*(*X*) = 1−*L*(*X*, *X*_0_, *σ*_0_) for a total of 18 fitting coefficients and *S*_res_(*Y*) = 0.018. Table 2 contains the final values for the 18 fitting coefficients and other data that will be discussed in the next paragraph.

First and foremost, the *S*_res_(*Y*) = 0.018 is sufficiently small for the purposes of simulating the electronic and optoelectronic behavior of semiconductor devices, and verifies the adequacy of the fitted function, Eq. (19), and its 18 coefficients given in Table 2. The value of *S*_res_(*Y*) = 0.018 corresponds to a relative residual standard deviation of about 10 % which is more than adequate for most microelectronic and optoelectronic computer simulations. Second, the condition of near-constancy of spacing of the intercepts *I_i_* is met because for the worst case of *x* = 0.30, |*a*_0_ + *a*_1_ *x*|*_x_*_=0.3_ = 0.593 is much greater than |*a*_2_ *x*^2^ + *a*_3_ *x*^3^|*_x_*_=0.3_ = 0.038. And third, the condition of near-constancy of the slopes *S_i_* is met because for the worst case of *x* = 0.30 |*s*_0_| = 1.0 is much greater than |*s*_1_ *x* + *s*_2_ *x*^2^ + *s*_3_ *x*^3^|*_x_*_=0.3_ = 0.199. All 10 of the coefficients in *B*(*X*) are significant because the absolute values of their estimated standard deviation ratios greatly exceed the nominal value of 2. The estimated standard deviation ratio is the estimated value of a fitting parameter divided by its estimated standard deviation. It gives the number of standard deviations by which a fitting parameter differs from zero. The nominal value of 2 is based on the 97.5 % point of the normal (Gaussian) distribution. In practice, coefficients with estimated standard deviation ratios less than two are considered to be statistically insignificant. Because the estimated standard deviation ratio of the intercept fitting parameter *a*_0_ is less than 2, *a*_0_ could be omitted from the final model with little effect. In fact, setting *a*_0_ = 0.0 gives the same value *S*_res_(*Y*) = 0.018 to within 10^−6^. The collective significance for the 17 remaining fitting parameters implies that the fitted function is parsimonious in its form and cannot be “trimmed” to a simpler form with fewer parameters or coefficients. For a further discussion of the above, we refer the reader to Draper and Smit [14].

Conventional non-linear fitting procedures usually do not contain regression analyses, transformations, and separable functions. As a consequence, they typically require more than 40 fitting coefficients for the data in Fig. 1, yield *S*_res_(*Y*) values for the data in Fig. 1 that are greater than 0.07. They therefore give analytic fits to the minority electron mobilities that may be of questionable value for use in device simulators.

The above closed-form fit of the data in Fig. 1 is of such precision that the fitted curves lie almost within the line-widths of the theoretical traces. The analytic expression in Eq. (19) now enables quantum mechanically based results, which required tens of hours of supercomputer time, to be readily and efficiently incorporated into commercial workstation-based simulations of HBTs.

The analytic fit in Eq. (19) is valid only within the ranges 0 ≤ *X* ≤ 4 and 0 ≤ *x* ≤ 0.30, and must not be used beyond this bounded two-dimensional space in which it is derived. Also, combining Eq. (19) with other transport models for mobilities, bandgaps, and effective intrinsic carrier concentrations that are derived from the interpretation of electrical measurements on the devices themselves may lead to incorrect descriptions of the electrical and optical behavior.

## 5. Engineering Significance

Using the above Eq. (19) and applying additional results from recent calculations [8,15] to microwave HBTs [16] for linear power amplifiers may suggest different design strategies to optimize HBT performance. The calculated changes in carrier densities of states (DOS), band edges, band offsets, effective carrier concentrations *n*_ie_, and carrier mobilities due to high dopant and carrier concentration effects in Ga_1−_ *_x_*Al*_x_*As are given in Refs. [8] and [15] at 300 K for mole fractions *x* of AlAs between 0.0 and 0.3, for donor densities *N*_D_ between 10^16^ cm^−3^ and 10^19^ cm^−3^, and for acceptor densities *N*_A_ between 10^16^ cm^−3^ and 10^20^ cm^−3^. Only one quantum mechanical theory is used to treat both sides of the Mott transition in these calculations. They give, with no fitting parameters to experimental measurements, an internally self-consistent description of carrier transport in Ga_1−_*_x_*Al*_x_*As/GaAs heterostructures for lasers, light emitting diodes, digital devices, and microwave devices. The predicted values for the distorted DOS, band edges, band offsets, *n*_ie_, and majority and minority mobilities differ significantly from those values found in many simulations of Ga_1−_*_x_*Al*_x_*As/GaAs heterostructures. Many simulators set *n*_ie_/*n*_i_ = 1 in Ga_1−_*_x_*Al*_x_*As for all *N*_D_ or *N*_A_, approximate *μ*_e_(p-type; *N*_A_) with *μ*_e_(n-type; *N*_D_ = *N*_A_), and assert that all mobilities are monotonically decreasing functions of the dopant density. However, Fig. 1 shows that a relative minimum exists for *μ*_e_(p-type; *N*_A_) and suggests that a different design strategy could be significant for linear HBT amplifiers in digital cellular phones. Because a relative minimum in the minority electron mobility as a function of the acceptor density exists, we have identified additional design considerations for HBT power amplifiers that would have not otherwise been known. The above relative minimum arises from dependencies of several competing scattering mechanisms on both the dopant and carrier densities. A reduction in the scattering of minority electrons off hole plasmons and the removal of majority hole carriers from minority carrier-majority carrier scattering due to the Pauli exclusion principle accounts for the relative minimum in the decade of 10^18^ cm^−3^.

If other parameters remain essentially the same and *N*_A_ increases from 6 × 10^18^ cm^−3^ to 6 × 10^19^ cm^−3^ then the following occurs:

1. the minority electron mobility increases by a factor of 2.5 [8],
2. the base transit time decreases by about a factor of 2.5, and
3. the base resistivity decreases by about a factor of 10 [17].

Combining these last three results into expressions from compact models for microwave HBTs predicts increases in operating frequencies of about 40 % and in figures of merits (maximum frequencies at unity gain) of about 300 %. These estimates are considered to be upper limits because more rigorous simulations depend on both processing and operating parameters whose choices are determined by the application.

## 6. Conclusions

We have constructed a two-dimensional closed-form analytic function for the minority electron mobilities in Ga_1−_*_x_*Al*_x_*As at 300 K that is a function of the mole fraction and acceptor doping density. All of the important scattering mechanisms including carrier-carrier and plasmon scattering are considered. The minority electron mobility from first-principles quantum mechanical calculations shows an interesting structure at high densities due to the reduction in plasmon scattering and the Pauli exclusion principle. The results are important for device modeling because of the need to have accurate values for minority mobilities which in turn allow improved design of HBTs for microelectronic and optoelectronic applications, for example, digital cellular phones and modulators in optical communications systems, respectively.

The general modeling approach given here may be used to:

1. generate close-form analytic expressions
2. eliminate interpolator look-up tables and
3. enable more efficient computer simulations for many microelectronics and optoelectronic applications such as obtaining expressions for carrier transport properties and extracting two-dimensional doping profiles from scanning capacitance microscopy measurements.

## Figures and Tables

**Table 1:** Two-dimensional array of data points from theoretical calculations of the normalized minority electron mobility^a^

*N*_A_	*x*						
10^16^ cm^−3^	0.00	0.05	0.10	0.15	0.20	0.25	0.30
1.00	5.742	5.136	4.552	4.003	3.500	3.051	2.658
2.00	5.106	4.584	4.081	3.606	3.171	2.779	2.434
3.00	4.718	4.244	3.788	3.357	2.961	2.604	2.288
5.00	4.225	3.810	3.411	3.034	2.686	2.371	2.092
7.00	3.903	3.524	3.161	2.818	2.501	2.214	1.959
1.00 × 10^1^	3.565	3.224	2.897	2.588	2.303	2.045	1.814
2.00 ×10^1^	2.926	2.654	2.394	2.148	1.921	1.715	1.530
3.00 ×10^1^	2.569	2.334	2.110	1.899	1.703	1.525	1.366
5.00 ×10^1^	2.145	1.953	1.771	1.599	1.441	1.296	1.165
7.00 ×10^1^	1.889	1.721	1.564	1.416	1.279	1.154	1.041
1.00 ×10^2^	1.643	1.499	1.364	1.239	1.123	1.016	0.9198
2.00 ×10^2^	1.258	1.150	1.050	0.9575	0.8725	0.7948	0.7241
3.00 ×10^2^	1.101	1.006	0.9191	0.8397	0.7670	0.7007	0.6403
5.00 ×10^2^	0.9936	0.9046	0.8249	0.7530	0.6880	0.6292	0.5760
7.00 ×10^2^	0.9901	0.8971	0.8152	0.7420	0.6764	0.6175	0.5647
1.00 ×10^3^	1.068	0.9601	0.8660	0.7832	0.7097	0.6449	0.5873
2.00 ×10^3^	1.539	1.372	1.227	1.098	0.9863	0.8881	0.8013
3.00 ×10^3^	1.964	1.746	1.556	1.390	1.243	1.115	1.002
5.00 ×10^3^	2.489	2.212	1.968	1.753	1.563	1.397	1.250
7.00 ×10^3^	2.692	2.392	2.125	1.892	1.687	1.506	1.347
1.00 ×10^4^	2.720	2.418	2.152	1.918	1.711	1.528	1.368

a *Y* = *μ*_e_(p-type; *N*_A_, *x*)/*μ*_ref_ for p-type Ga_1−_*_x_*Al*_x_*As, where the acceptor density is *N*_A_, the mole fraction of AlAs is *x*, and *μ*_ref_ = 1000 cm^2^/(V·s) [8].

**Table 2:** The 18 final fitting parameters for the minority electron mobility from Eq. (19)^a^

Intercept and slope fitting parameters	Estimated value	Estimated standard deviation	Ratio
*a*_0_	−0.000 05	0.1052 × 10^−3^	−0.48
*a*_1_	−1.976 36	0.3323 × 10^−2^	−0.59 × 10^+3^
*a*_2_	0.524 177	0.2716 × 10^−1^	19.0
*a*_3_	−0.339 191	0.5943 × 10^−1^	−5.7
*s*_0_	0.999 986	0.3411 × 10^−4^	2.9 × 10^+4^
*s*_1_	−0.061 328	0.1077 × 10^−2^	−57.0
*s*_2_	−1.530 36	0.8808 × 10^−2^	−1.7 × 10^+2^
*s*_3_	1.695 421	0.1927 × 10^−1^	88.0

Base-reference function fitting parameters			

*c*_10_	1.741 24	0.001 598	1100.
*c*_11_	−0.350 382	0.009 642	−36.
*c*_12_	−0.076 087	0.014 74	−5.2
*c*_13_	−0.038 110	0.007 677	−5.0
*c*_20_	68.654 8	0.103 7	660.
*c*_21_	−50.516 9	0.216 0	−230.
*c*_22_	12.841 4	0.002 999	4300.
*c*_23_	−1.106 75	0.011 30	−98.
*X*_0_	3.242 62	0.042 98	75.
*σ*_0_	0.259 828	0.009 315	28.

a *μ*_e_(p-type; *N*_A_, *x*) = *μ*_ref_exp{*I*(*x*) + *S*(*x*)[*w*_L_(*X*)*g*_1_(*X*) + (1 − *w*_L_(*X*))*g*_2_(*X*)]}, where *I*(*x*) = *a*_0_ + *a*_1_ *x* + *a*_2_ *x*^2^ + *a*_3_ *x*^3^, *S*(*x*) = *s*_0_ + *s*_1_ *x* + *s*_2_ *x*^2^ + *s*_3_ *x*^3^, *g*_1_(*X*) =*c*_10_ + *c*_11_ *X* + *c*_12_ *X*^2^ + *c*_13_ *X*^3^, *g*_2_(*X*) = *c*_20_ + *c*_21_ *X + c*_22_ *X*^2^ + *c*_23_ *X*^3^, and *w*_L_(*X*) = 1/{1 + exp[(*X − X*_0_)/*σ*_0_]} where the normalized-dimensionless acceptor density is *X* = log_10_ (*N*_A_/10^16^ cm^−3^), the mole fraction of AlAs is *x*, and *μ*_ref_ = 1000 cm^2^/(V·s). All of the fitting parameters are dimensionless. The ratio is the estimated value divided by its estimated standard deviation.

## Footnotes

1 Certain commercial equipment instruments or materials are identified in this paper to foster understanding. Such identification does not imply recommendation or endorsement by the National Institute of Standards and Technology, nor does it imply that the materials or equipment identified are necessarily the best available for the purpose.
